# Predicting Blood Glucose Concentration after Short-Acting Insulin Injection Using Discontinuous Injection Records

**DOI:** 10.3390/s22218454

**Published:** 2022-11-03

**Authors:** Baoyu Tang, Yuyu Yuan, Jincui Yang, Lirong Qiu, Shasha Zhang, Jinsheng Shi

**Affiliations:** 1 School of Computer Science (National Pilot Software Engineering School), Beijing University of Posts and Telecommunications, Beijing 100876, China; 2 Key Laboratory of Trustworthy Distributed Computing and Service, Ministry of Education, Beijing 100876, China

**Keywords:** deep neural network, deep learning, insulin efficacy prediction, blood glucose prediction

## Abstract

Diabetes is an increasingly common disease that poses an immense challenge to public health. Hyperglycemia is also a common complication in clinical patients in the intensive care unit, increasing the rate of infection and mortality. The accurate and real-time prediction of blood glucose concentrations after each short-acting insulin injection has great clinical significance and is the basis of all intelligent blood glucose control systems. Most previous prediction methods require long-term continuous blood glucose records from specific patients to train the prediction models, resulting in these methods not being used in clinical practice. In this study, we construct 13 deep neural networks with different architectures to atomically predict blood glucose concentrations after arbitrary independent insulin injections without requiring continuous historical records of any patient. Using our proposed models, the best root mean square error of the prediction results reaches 15.82 mg/dL, and 99.5% of the predictions are clinically acceptable, which is more accurate than previously proposed blood glucose prediction methods. Through the re-validation of the models, we demonstrate the clinical practicability and universal accuracy of our proposed prediction method.

## 1. Introduction

Diabetes mellitus is an increasingly common chronic disease, which is mainly characterized by hyperglycemia and often induces multiple complications [1]. Hyperglycemia is also commonly encountered in the intensive care unit (ICU). Stress hyperglycemia (SH) often occurs in critically ill patients, even without a past diagnosis of diabetes [2,3,4]. Numerous observational studies demonstrate that elevated blood glucose is significantly associated with increased ICU mortality [5,6,7,8,9,10], and the aggressive correction of hyperglycemia with exogenous insulin can reduce the morbidity and mortality of ICU patients [11,12,13,14,15,16]. Continuously tracking blood glucose values is the best way to understand the effects of insulin on patients’ blood glucose concentrations. Point sample test and continuous glucose monitoring (CGM) are currently the most commonly used blood glucose detecting methods [17]. The point sample test requires patients to draw blood with a lancet several times daily. Such discrete tests are prone to miss hyperglycemic or hypoglycemic events. The CGM system uses a microsensor inserted beneath the skin and held for a period to assess glucose levels in tissue fluids. However, limitations in detection accuracy and sensor lifetime make it still necessary for patients to order point sample tests for continuous calibration. The inconvenience, discontinuity, and limited accuracy of these two blood glucose detection methods have brought great hidden dangers to patients in clinical practice. Therefore, accurately predicting the blood glucose concentrations after exogenous insulin injections is of great significance for maintaining the stability of blood glucose, preventing the side effect of hypoglycemia, and reducing the mortality of patients in clinical practice. In addition, the accurate prediction of insulin efficacy is also a necessary basis for the development of automated medical technologies, such as intelligent blood-glucose-monitoring devices, automatic insulin-delivery devices, and intelligent patient-monitoring systems.

The deep neural network is an important branch of artificial intelligence. Related concepts and algorithms have been proposed since the 1960s [18,19,20,21,22,23]. With the improvement of algorithms and the development of computer hardware, deep neural networks have flourished and been widely used in the past 20 years. In 2006, Hinton proposed deep belief nets (DBN) [24], which used a greedy unsupervised training method to solve the problem of handwritten digit recognition and achieved good results. In 2011, researchers from Microsoft proposed a pre-trained deep neural network hidden Markov model hybrid architecture (DNN-HMM) [25] that greatly reduced the error rate of speech recognition. Alex Krizhevsky proposed AlexNet in 2012 [26], reducing the Top5 error rate of ImageNet classification from 26% to 15%. Google proposed FaceNet in 2014 [27], achieving 99.63% test accuracy on the Labeled Faces in the Wild (LFW) dataset. Microsoft built a 152-layer residual neural network(ResNet) [28] in 2015, reducing the classification error rate of ImageNet to 3.57%. In 2016, AlphaGo [29], which integrates Monte Carlo simulation and value and policy networks, defeated the world Go champion. There are great expectations on how this technology can be applied to healthcare and clinical practice [30,31,32]. In recent years, the deep neural network has also shown its feasibility in many medical applications, such as automatic diagnosis [33,34,35], medical image interpretation and processing [36,37,38,39], risk prediction [36,40,41], and toxicity prediction [42,43].

In the past five years, studies on predicting blood glucose concentrations have also been reported [44,45,46,47]. It has been observed that convolutional neural network (CNN), recursive neural network (RNN), and the variations of these network models are commonly used in prediction studies, and the prediction results with low root mean square error (RMSE) can be obtained. A study by K. Li et al. [48] presented a deep learning algorithm for glucose prediction using a multi-layer convolutional recurrent neural network (CRNN). The model was primarily trained on a simulated dataset of 10 cases generated from the UVA/Padova simulator and a clinical dataset from 10 real patient cases, each containing continuous glucose readings, insulin bolus injections, and carbohydrate data for six months. Their predictive model had leading accuracy for real patient cases (RMSE = 21.07 mg/dL for 30 min and RMSE = 33.27 mg/dL for 60 min on average). S. Mirshekarian et al. [49] constructed a recursive neural network model that uses long short-term memory (LSTM) units to train a physiological model of blood glucose. The LSTM network was trained and evaluated separately on the dataset of five different patients, containing approximately 400 days of records on each patient’s blood glucose values, insulin doses, and dietary intake. The networks trained separately for each specific patient could achieve competitive predicting accuracy (RMSE = 21.4 mg/dL for 30 min, RMSE = 38.0 mg/dL for 60 min on average). H.V. Dudukcu et al. [50] created a consolidated model using gated recurrence unit (GRU) to predict blood glucose with the OhioT1DM dataset. The RMSE values obtained in experimental studies suggested that the consolidated model trained using all patients’ data provides better results than the individual model trained specifically for each patient.

Although there have been many related studies using deep neural networks to predict blood glucose concentrations (shown in Appendix A Table A1), most of these studies have limitations in the scope of application. Most of the proposed deep neural networks are trained using continuous recording data from diabetic patients [45,46,47,48,49,50,51,52], including the blood glucose values recorded by CGM and other recordings of some correlative events. Since the network training requires continuous records lasting tens or even hundreds of days, the acquisition of the datasets is very difficult. Some studies [48,49,52] also require patients to record multiple daily information manually for hundreds of consecutive days, such as insulin injection events, dietary events, exercise events, and psychological stress, resulting in many missing and erroneous values in the dataset with fewer usable data. For the above reasons, the networks are always trained with datasets only from a small number of clinical patients [45,47,48,49,50,51,52] (mostly below 30 patients) or even with simulated dataset from virtual patients [44,47,48]. The networks trained using datasets from a few patients can only simulate the physiological environment of a few specific people, and have very limited accuracy in predicting for patients that are not involved in the training data, which leads to the difficulty of predicting blood glucose after insulin injection with deep neural networks, and cannot be widely applied in clinical practice for the patients without continuous data records.

In this study, we try to explore whether the three commonly used deep learning regression models, including fully connected neural network (FCN), progressive neural network (CNN), and long short-term memory network (LSTM), could predict the effect of short-term insulin doses on blood glucose concentrations without the need for continuous past records. We extract independent discontinuous short-acting insulin-injection events occurring in the ICU from the Medical Information Mart for Intensive Care-IV (MIMIC-IV) database [53], and match each insulin injection record to the glucose values that triggered and related to the insulin treatment. Moreover, we also integrate some clinical characteristics that have been proven to be associated with insulin efficacy as predictive references, including the patient’s age, gender, weight, ethnicity, blood pressure, and renal function indexes [54,55,56,57,58,59,60]. Using these atomic records of discrete insulin injection events to train deep neural networks, and taking these demographic characteristics and basic examination results of ICU patients as the network input, we construct various deep neural networks with different architectures to predict the blood glucose concentrations of ICU patients about 4 h after short-acting insulin injections. We re-validate the predictive accuracy of the network models using the K-fold test and the Chi-square test to demonstrate the universal accuracy of our proposed networks.

## 2. Proposed Method

### 2.1. Dataset Extraction and Integration

We extracted our dataset from the MIMIC-IV database version 1.0. MIMIC-IV is an extensive, freely available database containing real medical information during hospital stays and ICU stays for patients admitted to a tertiary academic medical center in Boston, MA, USA. The data include the patient demographics, bedside monitoring of vitals, administration of medications, results of laboratory measurements, as well as diagnoses, procedures, and prescriptions for billing.

The primary objectives of our data extraction are as follows:

1. Extract discontinuous short-acting insulin bolus injections occurring in the ICU from the electronic medical records in the MIMIC-IV database.
2. Match the blood glucose values before insulin injection and around the peak time of insulin efficacy for each insulin injection event.
3. Query and match the relevant characteristic values for each insulin injection event as the prediction basis.
4. Remove all missing values and possible erroneous values.

**Figure 1:**
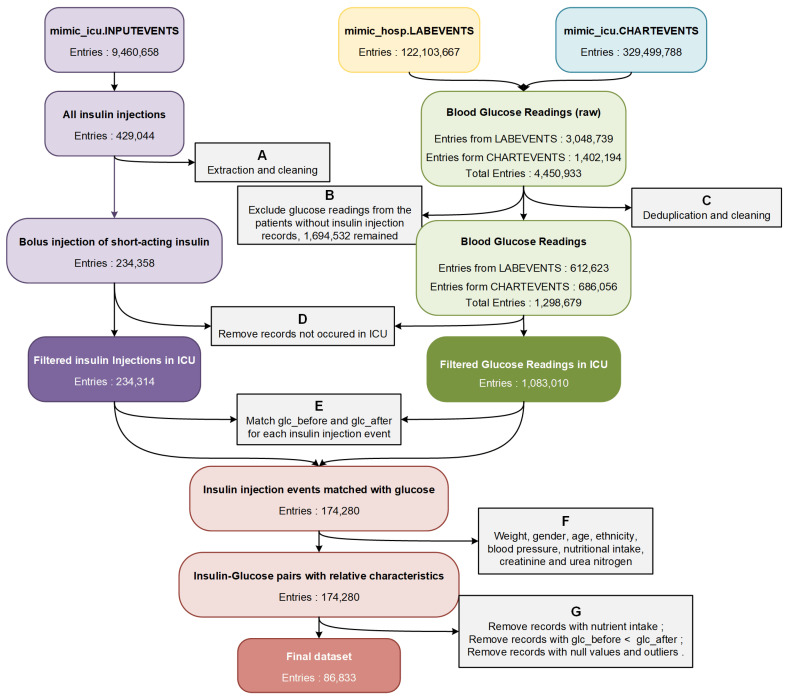
The overview process of dataset extraction.

For that purpose, we formulated data filtering and integration rules based on data analysis, clinician recommendations, and physiological and pharmacological standards. The overall process of data extraction is shown in Figure 1.

Firstly, we extracted the records of short-acting insulin injection events (containing regular insulin, Humalog, and Novolog) from the table mimic_icu.INPUTEVENTS. After removing erroneous and incomplete data, we obtained 234,358 bolus injection records of short-acting insulin from 24,750 patients and 31,920 ICU stays (step A in Figure 1). Then we extracted the blood glucose values measured by laboratory chemistry analyzers from table mimic_hosp.LABEVENTS, and the blood glucose values measured by bedside fingerstick glucometers from table mimic_icu. CHARTEVENTS (4,450,933 in total). Posteriorly, glucose data from patients who did not receive any insulin injection were excluded (step B in Figure 1). After removing duplicates, errors, and outliers, we obtained a total of 1,298,679 blood glucose test results (step C in Figure 1). We matched the occurrence time of all blood glucose readings and insulin injection events to the ICU admission records from table mimic_icu.ICUSTAYS, and all events that did not occur in the ICU were removed (step D in Figure 1).

Secondly, to predict the effect of insulin doses on blood glucose concentrations, we tried to match patients’ blood glucose values before and after insulin injection (glc_before and glc_after) for each insulin injection event according to time (step E in Figure 1). We calculated the time interval between each insulin injection and the closest blood glucose detection before and after the injection event (timediff_before and timediff_after).

**Figure 2:**
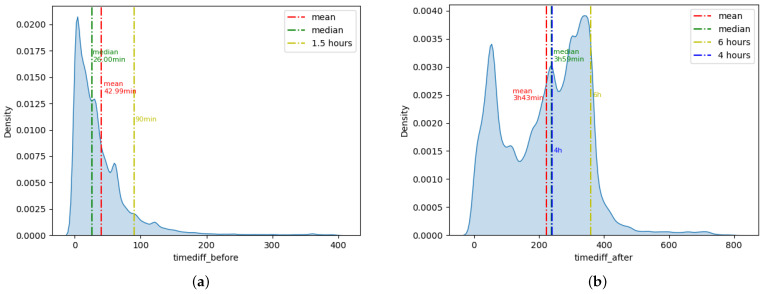
(**a**) Probability density diagram of timediff_before. (**b**) Probability density diagram of timediff_after.

Figure 2a shows the density distribution of timediff_before. A total of 89.36% of the insulin injection events had a blood glucose record within 90 min before the insulin injection. Therefore, we selected the blood glucose value closest to the insulin injection event as glc_before from all blood glucose records within 90 min before and 5 min after the insulin injection. If there were no blood glucose records within 90 min before insulin injection, we would extend the backward search time and select the closest blood glucose results within 10 min after insulin injection as glc_before.

Figure 2b shows the density distribution of timediff_after. The mean value of timediff_after was 3 h 43 min, and the median was 3 h 59 min. A total of 92.29% of insulin injection events have blood glucose records within 6 h after the insulin injection. Considering that the peak effect of short-acting insulin is about 4 h after injection, we selected the blood glucose value closest to 4 h and within 6 h after insulin injection as glc_after. Finally, we obtained 174,280 insulin injection events that were successfully matched to glc_before and glc_after.

In addition, to further improve the prediction accuracy of the insulin effect, we extracted many related characteristics from the MIMIC-IV database (step F in Figure 1). We obtained the patients’ weight and nutritional intake from table mimic_icu.INPUTEVENTS, patients’ ethnicity from table mimic_core.ADMISSIONS, patients’ gender and age from table mimic_core.PATIENTS, the average systolic and diastolic blood pressure (sbp and dbp) within two hours around insulin injection from table mimic_icu.CHARTEVENTS, and the values of creatinine and blood urea nitrogen (bun) from table mimic_hosp.LABEVENTS. Since the total amount of patient sugar intake could not be accurately measured according to the records from the MIMIC-IV database, we removed all the insulin injection records with nutrient solutions ingesting between the two blood glucose measurements before and after insulin injection. We also removed all the insulin injection records with glc_before less than glc_after to avoid the unknown nutrient intake affecting our prediction of the insulin efficacy. Finally, after removing all records with missing values and outliers, we obtained 86,833 insulin injection records as the dataset for predicting the effect of insulin doses on patients’ blood glucose concentrations (step G in Figure 1).

### 2.2. Neural Network Training and Hyperparameter Tuning

For data preprocessing, we performed one-hot encoding for two discrete input characteristics (gender and ethnicity). For other numerical input characteristics, we performed min-max normalization and added the square and root of the normalized values into the input. We ended up with a 31-dimensional input dataset for all deep neural networks.

We trained three commonly used numerical regression neural networks for glucose prediction, including FCN, CNN and LSTM. We trained all the network models with different architectures using 80% of our dataset as the same training set. To explore the model architectures with high prediction accuracy, we generated network architectures with different depths and widths for these three kinds of neural network models.

We first tried to train FCNs with different numbers of fully connected layers (FL). When the number of network layers is less than 10, the network prediction accuracy improves with the increase in the network depth, indicating that there is still room for improvement. The prediction accuracy of the 10-layer and 11-layer networks is very close, the accuracy of 12-layer networks has a notable increase, and the 13-layer networks show a decrease in the testing accuracy and have an overfitting tendency. Through experiments, we found that CNN and LSTM networks have similar rules in the number of network layers and the prediction accuracy, so most of the layer numbers we tried were between 10 and 12. We also performed similar experiments to decide the width of the network layers, and we chose the layer size that improves prediction accuracy without causing overfitting while minimizing the computational cost. We tried to use different numbers of convolution layers (CL) and pooling layers (PL) in CNN. For the LSTM networks, we experimented with different numbers of LSTM layers and dropout layers. The complete architectures of the 13 networks with the highest prediction accuracy (RMSE below 16.90 mg/dL) are shown in the Appendix A in Table A2.

The kernel initializer for all Dense layers was RandomNormal, and for all Conv1D layers and LSTM layers was GlorotUniform. The bias initializer for all layers was Zeros. ReLU was used as the activation function of all the network layers. We used the Adam optimizer to update the weights of the networks. The learning rate of the Adam optimizer was 0.001, the beta1 was 0.9, and the beta2 was 0.999. Mean square error (MSE) was used as the loss function in the training process. Therefore, the goal of all the network training processes is to generate **F** that minimizes the MSE:

$$argmin\frac{1}{n}\sum_{i=1}^{n}{\left(F\left(x_{i}\right)-y_{i}\right)}^{2}$$

The training epochs of deep neural networks are also important for prediction accuracy. To prevent insufficient training, we did not use the setting of earlystop. We tried different training epochs in 500 epochs increments. With the increase in training epochs, the prediction accuracy of the training set will gradually increase, while the prediction accuracy of the testing set will initially increase and then decrease. The decreased testing accuracy indicates that the network is overfitting to the training set, and the network training should be stopped. The training epochs with the highest test accuracy of each model are finally picked, as shown in the Appendix A in Table A2.

**Figure 3:**
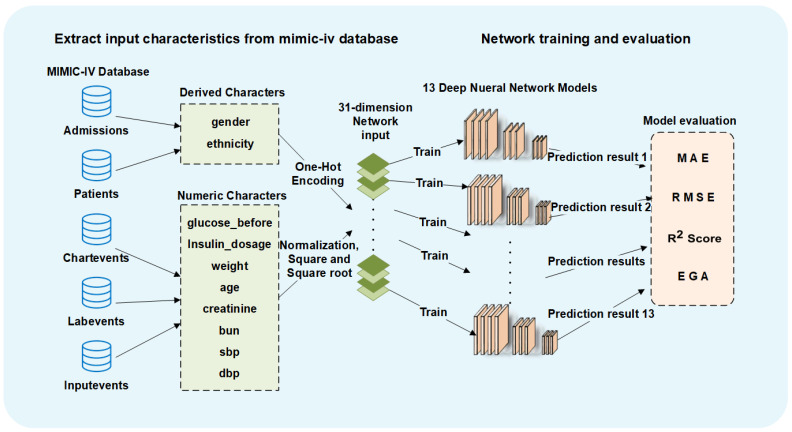
The overview process of the predicting framework.

All the networks were trained with the Python Keras framework on four NVIDIA TITAN Xp GPUs with 12 G memory size. To maximize the throughput of the utilization machine, we set the training batchsize to 1000. Figure 3 shows the complete process of our predicting methods.

### 2.3. Network Evaluation Metrics

We used the following four metrics to measure the prediction accuracy of all network models. (*y* represents the actual blood glucose value after insulin injection, $\hat{y}$ represents the predicted blood glucose value after insulin injection, $\bar{y}$ represents the average value of *y*, and *n* represents the total number of the testing set):

1. MAE: mean absolute error, which is calculated as the sum of absolute errors divided by the sample size. Lower values indicate better model fitting and more accurate prediction.
  $$MAE=\frac{1}{n}\sum_{i=1}^{n}\left|y_{i}-{\hat{y}}_{i}\right|$$
2. RMSE: root mean square error, which is the square root of the average squared errors. Lower values indicate better model fitting and more accurate prediction.
  $$RMSE=\sqrt{\frac{1}{n}\sum_{i=1}^{n}{\left(y_{i}-{\hat{y}}_{i}\right)}^{2}}$$
3. R2_Score: The coefficient of determination, denoted $R^{2}$ or $r^{2}$, can represent the proportion of the dependent variable that is predictable from the independent variables. Higher values indicate better model fitting and more accurate prediction.
  $$R^{2}=1-\frac{\sum_{i=1}^{n}{\left(y_{i}-{\hat{y}}_{i}\right)}^{2}/n}{\sum_{i=1}^{n}{\left(y_{i}-{\bar{y}}_{i}\right)}^{2}/n}=1-\frac{MSE}{Variance\;of\;y}$$
4. EGA [61]: Clarke error grid analysis, to quantify clinical accuracy of the predicted blood glucose values by subdividing the prediction results into five zones. The prediction values located in Zone A represent accurate predictions, and values in Zone B are acceptable glucose results. Values in Zone C may prompt unnecessary corrections. Values in Zone D indicate a dangerous failure to detect and treat. Values in Zone E represent erroneous treatment. More prediction results located in Zone A and Zone B indicate better model fit.

MAE, RMSE, and R2_ Score are all common metrics for measuring the performance of regression learning models. EGA is one of the “gold standards” for determining the clinical accuracy of blood glucose prediction. These four evaluation metrics provide us with different helpful information. MAE is a linear score, meaning that all individual differences are equally weighted in the average and can visually represent the absolute prediction error. In comparison, since the errors are squared before being averaged in the calculation process of RMSE, RMSE gives relatively high weight to the larger errors and is more sensitive to outliers, which means that RMSE is most useful when significant errors are especially undesirable. Unlike MAE and RMSE for calculating offset distance, R2_ Score indicates how well the predictor variables can explain the variation in the response variable. R2_ Score is conveniently scaled between 0 and 1, independent of the actual value of the regression task, which is obviously easier to interpret and compare across different regression tasks. As a widely recognized indicator, EGA can provide a reference for the clinical reliability of our prediction network.

Our study is dedicated to predicting blood glucose concentrations after short-acting insulin injections. Since our prediction results will serve as an important reference for clinicians or intelligent monitoring devices in determining insulin doses, our prediction network must avoid large prediction errors that may affect the clinical treatment. From the perspectives of clinical accuracy, safety, and reliability, we choose RMSE, which is more sensitive to large prediction errors, as the primary metric to compare the prediction accuracy of all network models, and use the other evaluation metrics as auxiliary references. Most previous glucose prediction studies have similarly used RMSE as the primary assessment criterion [45,46,47,48,49,50,52].

### 2.4. Machine Learning for Comparison

Regression prediction is one of the most common applications of machine learning. We used scikit-learn to build and train five commonly used regression machine learning models: support vector regression (SVR), K-nearest neighbors regression (KNN), classification and regression tree (CART), random forest regression (RF), and extreme gradient boosting regression (XGBoost). Scikit-learn is a Python module that integrates various state-of-the-art machine-learning algorithms for solving medium-scale supervised and unsupervised problems [62].

### 2.5. Model Retest

In order to verify the universal effectiveness of our network models and avoid the evaluation bias caused by the single testing set, we used K-fold cross validation [63] to re-validate the prediction accuracy of each network, which is a typical method for the reliability assessment of deep neural networks.

In our study, we relied on 5-fold cross validation to retrain and retest the 13 most accurate deep neural network models. We divided the entire dataset into five non-intersecting parts on average. For each training, we take four of the data parts as the training set and the remaining one as the testing set. After training and testing each model five times, all instances of the entire dataset were tested for each network model, and we obtained the mean prediction accuracy of the five testing sets for each network model. Furthermore, we performed the Chi-squared test [64] on the predicted RMSE values of the 13 networks on the 5 testing sets to prove that the networks have universal accuracy in predicting different testing sets.

## 3. Experiment Results

### 3.1. Dataset Extracted from MIMIC-IV Database

From 244,000 short-acting insulin bolus injection events from the MIMIC-IV database, through data screening, glucose matching, and characteristic integration, we obtained 86,833 independent insulin injection events that occurred in the ICU without missing values. These insulin injection records and the relevant information come from 25,168 ICU admissions of 20,426 adult patients. We used 80% of the entries in the dataset as the training set for deep neural networks, and the remaining 20% were used as the testing set.

Each insulin injection record contains 11 items, including the patient’s blood glucose before and after insulin injection, injected insulin dose, gender, age, weight, ethnicity, creatinine, blood urine nitrogen, systolic blood pressure, and diastolic blood pressure. The blood glucose before insulin injection is the closest glucose detection result to the injection event within 90 min before and 10 min after insulin injection. The blood glucose after insulin injection is determined according to the blood glucose detection results closest to 4 h after each injection event, which is the peak time of the efficacy of short-acting insulin. Creatinine and blood urine nitrogen are the basic vital signs of ICU patients, which are the main indicators reflecting the renal function of patients. Table 1 summarizes the statistical descriptions of 11 characteristics of the entire dataset that we created for this study, including the maximum, minimum, average, standard deviation and median of 9 digital characteristics, and the distribution of 2 discrete characteristics.

### 3.2. Deep Neural Networks and Performance Evaluation

In contrast to previous studies on blood glucose prediction shown in Appendix A Table A1, our study is not aimed at predicting the real-time blood glucose concentrations in the daily life of diabetic patients, but focuses on predicting the clinical effect of insulin dose on blood glucose in each insulin injection event. Most previous studies [45,47,48,50,52] require the relevant records of patients from tens of consecutive days for network training, and the trained models cannot predict the blood glucose for any patient without a long-term blood glucose history. However, in our study, we regarded each insulin injection as an independent atomic event unrelated to the patients’ historical state, and used the patients’ real-time characteristics at the time of insulin injection to predict the blood glucose after the short-acting insulin injection.

We built three kinds of deep neural networks, including FCN, CNN, and LSTM, to atomically predict the ICU patients’ blood glucose concentration after the short-acting insulin injection. We tried dozens different network model architectures to explore whether these three commonly used numerical regression networks can be used for glucose prediction based on discrete insulin injection records and tried to find network models with high accuracy. The same training set was used to train all network models and the same testing set for network evaluation. The testing results of each network were evaluated using MAE, RMSE, and R2_Score, which are the common metrics to measure the prediction accuracy. Furthermore, we used Clarke error grid analysis (EGA) to evaluate the network performance, which is dedicated to measuring the accuracy of blood glucose predictions.

**Figure 4:**
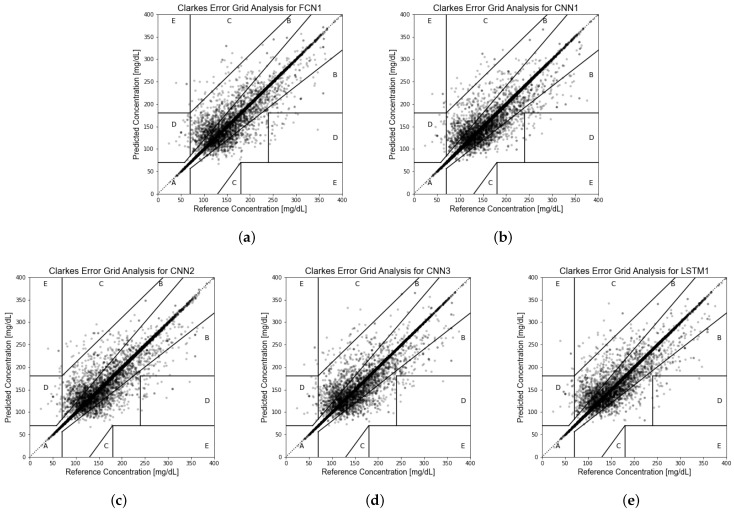
(**a**) Clarke Error Grid Analysis of model FCN1. (**b**) Clarke Error Grid Analysis of model CNN1. (**c**) Clarke Error Grid Analysis of model CNN2. (**d**) Clarke Error Grid Analysis of model CNN3. (**e**) Clarke Error Grid Analysis of model LSTM1.

After experiments and comparisons, we obtained 13 network architectures with excellent prediction accuracy (RMSE less than 16.9 mg/dL). The detailed architectures of the 13 networks are shown in Appendix A Table A2, and the testing accuracy of these 13 network models is shown in Table 2. As in previous studies [45,46,47,48,49,50,52], taking RMSE as the primary evaluation criterion, the testing RMSEs of the five most accurate networks are all about 16 mg/dL, and the smallest RMSE is as low as 15.82 mg/dL. The accuracy of predicting using stochastically independent insulin injection records in this study is much better than that of previous studies [45,46,47,48,49,50,52] (RMSE more than 20 mg/dL, shown in Appendix A Table A1) predicted by continuous glucose monitoring data. Clarke error grid analysis of the prediction results of the five most accurate models shows that about 94% of the predicted values are located in Zone A of the error grid, and 5.5% are in Zone B, which means that over 99.5% of testing results could be regarded as being clinically acceptable. The Clarke error grid of the prediction results of the testing set for these five network models with the lowest RMSE is shown in Figure 4.

The testing accuracy of our proposed models demonstrates that the three most commonly used deep neural regression models (FCN, CNN, and LSTM) can accurately predict the blood glucose concentration after any atomically independent insulin-injection event without requiring any long-term relevant records. Our proposed method makes blood glucose prediction more effective, convenient and feasible in clinical practice.

### 3.3. Comparison with Machine Learning

To demonstrate the superiority of deep neural networks, we tried to use several machine learning models for blood glucose prediction. We trained five nonlinear regression machine learning models, including SVR, KNN, CART, RF, and XGBoost. The five models were trained using the same training set as the deep neural networks, and the same testing set was used for accuracy evaluation. Table 3 shows the prediction accuracy of the five machine learning models used for comparison. Among the five machine learning models, RF has the lowest RMSE and the highest R2_Score (RMSE = 17.07 mg/dL, R2_Score = 0.8869), and the CART model has the lowest MAE (MAE = 5.97 mg/dL). RF also performs best in EGA, with approximately 92.80% of the prediction results located in Zone A and Zone B. The comparison results show that all of the 13 individual neural networks perform better than the best machine learning models in all accuracy evaluation metrics.

It is worth discussing that the RMSE of RF is very close to our proposed deep neural network models, but its MAE is much higher. RF is an ensemble learning method based on decision trees, which integrates the prediction results of many decision trees by regressing the average prediction value. Since RF averages the predictions of multiple CART models, RF has a significant effect in reducing variance, which results in better testing RMSE and R2_Score. However, the MAE value of our RF model is as high as 8.29 mg/dL, which indicates that the RF model tends to overfit during the training process. In pursuit of the accurate prediction of some noise data and reducing the RMSE, which is sensitive to outliers, RF sacrifices the absolute prediction error of the overall dataset in the training process.

### 3.4. Test–Retest Reliability

We retested 13 deep neural networks using 5-fold cross validation to verify the accuracy of the network models. The 86,833 records in the dataset were randomly divided into five parts called folds, and all of the folds have instances of equal size (17,366 for each fold). Each model was trained and tested five times, each time using one part as the testing set and the other four parts as the training set. The final prediction accuracy of the 5-fold cross validation for each network model was the average test performance of the five parts, which is presented in Table 4. For all the deep neural network models proposed in this study, the RMSE values after retesting does not increase by more than 0.5 mg/dL, demonstrating that our models are generally effective in predicting the effect of insulin dose on blood glucose for ICU patients.

We performed the Chi-squared test on the RMSE values of each testing fold for these 13 networks. The RMSE values of 13 models against five testing folds and the *p*-value of the Chi-squared test are shown in the Appendix A in Table A3. The *p*-value of the Chi-squared test is greater than 0.995, indicating that there is almost no statistical difference in the prediction accuracy for the five different testing sets, which further proves that the prediction results of the proposed models are generally accurate.

## 4. Conclusions

In this study, we developed 13 deep neural networks with different architectures, using 10 related characteristics as the network input to atomically predict the blood glucose concentrations of ICU patients after stochastically short-acting insulin injections without requiring any long-term history records..

We extracted 86,833 short-acting insulin bolus injection records discretely occurring in the ICU from the MIMIC-IV dataset, each record containing 11 characteristics associated with insulin efficacy. Using these records as the training and testing sets, we trained 13 deep neural network models with different architectures (shown in the Appendix A in Table A2). All of these 13 networks achieve good prediction accuracy. The testing RMSEs of these 13 deep neural networks are all below 16.90 mg/dL, reaching a minimum of 15.82 mg/dL, which is superior to five machine learning regression models (shown in Table 3) and the methods proposed in previous blood glucose prediction studies (shown in Appendix A Table A1.) The predicting EGA of these 13 network models shows that more than 99.5% of the prediction results are clinically acceptable.

In order to prevent the evaluation bias of model accuracy caused by the single testing set, we retested the 13 individual network models using 5-fold cross validation, and we performed the Chi-squared test on the prediction accuracy of the five different testing sets. The retesting results demonstrate that the network architecture proposed in this study is universally accurate in predicting the effect of insulin dose on blood glucose values.

In summary, our study demonstrates that it is a feasible, effective, and accurate method to atomically predict the effect of insulin doses on glucose concentrations using deep neural networks and discontinuous insulin injection records. The blood glucose concentration predicted by the deep neural networks has reference significance in clinical drug dosage. The various deep neural network architectures proposed in this study provide a reference for subsequent studies related to automatic insulin injection and continuous glucose monitoring. Our network models also provide basic simulation environments for further study on insulin dose recommendations using deep reinforcement learning.

## Figures and Tables

**Table 1:** Statistical description of the 11 characteristics in the dataset.

Characteristics ^1^	Maximum	Minimum	Mean ± Standard Deviation	Median
Blood glucose before insulin (mg/dL)	399.00	59.00	198.49 ± 56.45	184.00
Blood glucose after insulin (mg/dL)	398.00	17.00	155.08 ± 50.58	146.00
Insulin dose (unit)	35.00	0.10	4.69 ± 3.60	4.00
Age	91.00	18.00	65.35 ± 13.76	67.00
Weight (kg)	587.00	23.90	86.86 ± 25.08	83.70
Systolic blood pressure (mmHg)	228.00	39.00	119.27 ± 19.44	116.50
Diastolic blood pressure (mmHg)	156.00	14.70	60.2 ± 12.59	58.75
Creatinine (mg/dL)	22.60	0.10	1.61 ± 1.50	1.10
Blood urea nitrogen (mg/dL)	283.00	2.00	34.26 ± 26.44	25.00
Gender (num/%) ^2^	Female		Male	
	33,942/39.09%		52,891/60.91%	
Ethnicity (num/%) ^3^	American Indian, Asian, Black, Hispanic(Latino), White, Others (Unknown)			
	205, 2285, 8549, 3676, 58,136, 13,982/0.24%, 2.63%, 9.85%, 4.23%, 66.95%, 16.10%			

The table summarizes the basic statistical characteristics of the dataset used in this study. ^1^ Each record contains 11 characteristics with different units of measurement. The table contains the minimum, maximum, mean, median and standard deviation of nine numerical characteristics, and the distribution of two discrete characteristics. ^2^ Distribution of patients’ gender for all entries in the dataset. ^3^ Distribution of patients’ ethnicity for all entries in the dataset, including six categories.

**Table 2:** The prediction accuracy of 13 deep neural networks.

Network_id	RMSE (mg/dL) ^1^	MAE (mg/dL) ^2^	R2_Score ^3^	EGA (A%_B%_C%_D%_E%) ^4^
FCN1	16.19	5.27	0.8982	94.06%_5.48%_0.17%_0.27%_0.02%
FCN2	16.56	5.79	0.8935	94.00%_5.53%_0.13%_0.30%_0.04%
CNN1	16.06	5.56	0.8999	94.07%_5.51%_0.16%_0.22%_0.04%
CNN2	16.17	5.55	0.8984	93.97%_5.59%_0.12%_0.29%_0.03%
CNN3	16.24	5.06	0.8977	94.03%_5.53%_0.17%_0.27%_0.03%
CNN4	16.24	5.21	0.8976	94.26%_5.27%_0.10%_0.34%_0.02%
CNN5	16.29	5.09	0.8969	94.03%_5.51%_0.16%_0.28%_0.02%
CNN6	16.54	6.40	0.8937	93.71%_5.78%_0.16%_0.32%_0.03%
CNN7	16.29	5.66	0.8970	93.99%_5.59%_0.12%_0.26%_0.03%
LSTM1	15.82	5.17	0.9027	94.60%_4.95%_0.17%_0.25%_0.03%
LSTM2	16.82	7.47	0.8902	94.03%_5.49%_0.19%_0.27%_0.02%
LSTM3	16.88	10.63	0.8894	93.95%_5.53%_0.18%_0.31%_0.03%
LSTM4	16.40	5.35	0.8956	93.95%_5.58%_0.13%_0.31%_0.03%

^1^ Root mean square error, the standard deviation of the prediction error, lower values indicate better model fit. ^2^ Mean absolute error, the arithmetic average of the absolute errors, lower values indicate better model fit. ^3^ The coefficient of determination, the proportion of dependent variables that can be predicted through regression relationship; larger values indicate better model fit. ^4^ Clarke error grid analysis; the more values that appear in Zones A and B indicate better model fit.

**Table 3:** The prediction accuracy of five machine learning models.

Model Type	RMSE (mg/dL)	MAE (mg/dL)	R2_Score	EGA (A%_B%_C%_D%_E%)
SVR	34.04	25.43	0.5504	72.65%_25.60%_0.66%_0.97%_0.13%
KNN	22.20	9.75	0.8086	89.02%_9.98%_0.23%_0.69%_0.08%
CART	20.04	5.97	0.8441	92.74%_6.56%_0.26%_0.33%_0.10%
RF	17.07	8.29	0.8869	92.80%_6.58%_0.11%_0.48%_0.03%
XGBoost	30.15	23.08	0.6472	77.00%_21.65%_0.26%_1.04%_0.05%

**Table 4:** The average prediction accuracy of 5-fold cross validation of 13 neural network models.

Network_id	RMSE (mg/dL)	MAE (mg/dL)	R2_Score	EGA (A%_B%_C%_D%_E%)
FCN1	16.40	5.34	0.8947	94.03%_5.50%_0.18%_0.27%_0.02%
FCN2	16.56	5.49	0.8926	94.21%_5.33%_0.12%_0.30%_0.04%
CNN1	16.33	5.67	0.8956	94.07%_5.44%_0.17%_0.29%_0.02%
CNN2	16.20	5.36	0.8973	94.02%_5.52%_0.16%_0.27%_0.03%
CNN3	16.45	5.71	0.8942	93.94%_5.56%_0.18%_0.28%_0.03%
CNN4	16.18	5.27	0.8975	93.97%_5.56%_0.17%_0.27%_0.02%
CNN5	16.30	5.18	0.8961	94.07%_5.47%_0.20%_0.23%_0.03%
CNN6	16.30	5.63	0.8961	94.07%_5.48%_0.16%_0.27%_0.03%
CNN7	16.32	5.58	0.8958	93.99%_5.55%_0.16%_0.27%_0.03%
LSTM1	16.53	5.39	0.8932	93.90%_5.58%_0.21%_0.28%_0.03%
LSTM2	17.06	7.48	0.8862	93.97%_5.49%_0.20%_0.31%_0.02%
LSTM3	17.15	6.34	0.8849	93.72%_5.73%_0.20%_0.29%_0.04%
LSTM4	16.61	5.26	0.8920	93.86%_5.66%_0.20%_0.25%_0.03%

## Data Availability

The MIMIC-IV database is openly available in https://doi.org/10.13026/s6n6-xd98 (accessed on 23 December 2021).

## Appendix A. Appendix Tables

**Table A1:** Summary of previous studies addressing blood glucose prediction using deep nueral networks: model type, number of participants in the cohort, mean number of monitored days per patient, methods and frequency of blood glucose monitoring, whether continuous recording is required (C), whether manual record is required (M), prediction horizon (PH), test performance of the model, citation and publish year.

Model	Cohort	Days	Glucose Record	C	M	PH	Test Performance	Ref	Year
**Previous related studies:**									
FCN ^1^	100 VP ^2^	-	random simulation			after meal	reduce BGRI ^3^	[44]	2018
LSTM ^4^	1 T2D ^5^	30	by SMBG ^6^	**✓**		60 min	RMSE ^7^≈18.79 mg/dL	[45]	2019
LSTM	174 patients	2∼3	every 5 min by CGM ^8^	**✓**		30/60/120 min	RMSE≈19.44/22.86/24.48 mg/dL	[46]	2019
CNN ^9^	20 VP, 16 T1D ^10^	180	every 5 min by CGM	**✓**	**✓**	30/60 min	RMSE≈19.28/31.83 mg/dL	[47]	2020
CRNN ^11^	10 VP, 10 T1D	180	every 5 min by CGM	**✓**	**✓**	30/60 min	RMSE≈21.07/33.27 mg/dL	[48]	2020
LSTM	5 T1D	400	every 5 min by CGM	**✓**	**✓**	30/60 min	RMSE≈21.4/38.0 mg/dL	[49]	2017
GRU ^12^	12 T1D	56	every 5 min by CGM	**✓**		30 min	RMSE≈21.54 mg/dL	[50]	2021
FCN	25 T1D	1∼2	every 5 min by CGM	**✓**		30 min	PRED-EGA ^13^, accuracy < 95%	[51]	2017
FCN	27 patients	16	every 5 min by CGM	**✓**	**✓**	75 min	RMSE≈43.9 mg/dL	[52]	2011
**Best prediction model proposed in this study:**									
LSTM	20,426 patients	-	discrete record			about 4h	**RMSE≈15.82 mg/dL**	-	2022

^1^ Fully connected neural network. ^2^ Virtual patient. ^3^ Blood glucose risk index. ^4^ Long short-term memory network. ^5^ Type 2 diabetes. ^6^ Self-monitoring blood glucose device. ^7^ Root mean square error. ^8^ Continuous glucose monitoring devices. ^9^ Convolutional neural network. ^10^ Type 1 diabetes. ^11^ Convolutional recurrent neural network. ^12^ Gated recurrence unit. ^13^ Prediction-error grid analysis, an extension of the Clark error grid analysis.

**Table A2:** Detailed architecture of 13 different deep neural networks.

**network_id:**	FCN1	**layer number:**	12	**parameters number:**	6306945
**training epochs:**	7000	**layer type:**	Fully Connected Layer(units) ^1^		
**network architecture:**					
FCL(2048)-FCL(1536)-FCL(1024)-FCL(768)-FCL(512)-FCL(384)-FCL(256)-FCL(128)-FCL(64)-FCL(32)-FCL(16)-FCL(1)					
**network_id:**	FCN2	**layer number:**	10	**parameters number:**	914905
**training epochs:**	4000	**layer type:**	Fully Connected Layer(units)		
**network architecture:**					
FCL(1024)-FCL(512)-FCL(400)-FCL(256)-FCL(128)-FCL(72)-FCL(64)-FCL(32)-FCL(16)-FCL(1)					
**network_id:**	CNN1	**layer number:**	12	**parameters number:**	1040241
**training epochs:**	7000	**layer type:**	Convolutional Layer(filter, kernel_size) ^2^, Pooling Layer(pool_size) ^3^, Fully Connected Layer(units)		
**network architecture:**					
CL(16,3)-CL(64,3)-CL(128,3)-CL(256,3)-CL(512,3)-CL(256,3)-CL(128,3)-CL(64,3)-CL(16,3)-CL(8,3)-PL(3)-FCL(1)					
**network_id:**	CNN2	**layer number:**	12	**parameters number:**	5868897
**training epochs:**	4000	**layer type:**	Convolutional Layer(filter, kernel_size), Fully Connected Layer(units)		
**network architecture:**					
CL(32,3)-CL(64,3)-CL(128,3)-CL(64,3)-FCL(2048)-FCL(1024)-FCL(512)-FCL(256)-FCL(128)-FCL(64)-FCL(16)-FCL(1)					
**network_id:**	CNN3	**layer number:**	12	**parameters number:**	1040353
**training epochs:**	7000	**layer type:**	Convolutional Layer(filter, kernel_size),Fully Connected Layer(units)		
**network architecture:**					
CL(16,3)-CL(64,3)-CL(128,3)-CL(256,3)-CL(512,3)-CL(256,3)-CL(128,3)-CL(64,3)-CL(16,3)-CL(8,3)-CL(4,3)-FCL(1)					
**network_id:**	CNN4	**layer number:**	12	**parameters number:**	9381633
**training epochs:**	4000	**layer type:**	Convolutional Layer(filter, kernel_size), Fully Connected Layer(units)		
**network architecture:**					
CL(16,3)-CL(64,3)-CL(128,3)-FCL(2048)-FCL(1024)-FCL(512)-FCL(256)-FCL(128)-FCL(64)-FCL(32)-FCL(16)-FCL(1)					
**network_id:**	CNN5	**layer number:**	12	**parameters number:**	7753953
**training epochs:**	7000	**layer type:**	Convolutional Layer(filter, kernel_size),Fully Connected Layer(units)		
**network architecture:**					
CL(32,3)-CL(64,3)-CL(128,3)-CL(64,3)-FCL(2048)-FCL(1536)-FCL(768)-FCL(384)-FCL(128)-FCL(64)-FCL(16)-FCL(1)					
**network_id:**	CNN6	**layer number:**	12	**parameters number:**	1041937
**training epochs:**	7000	**layer type:**	Convolutional Layer(filter, kernel_size), Pooling Layer(pool_size), Fully Connected Layer(units)		
**network architecture:**					
CL(16,3)-CL(64,3)-CL(128,3)-CL(256,3)-CL(512,3)-CL(256,3)-CL(128,3)-CL(64,3)-CL(16,3)-PL(3)-FCL(32)-FCL(1)					
**network_id:**	CNN7	**layer number:**	11	**parameters number:**	1047777
**training epochs:**	4000	**layer type:**	Convolutional Layer(filter, kernel_size),Fully Connected Layer(units)		
**network architecture:**					
CL(32,3)-CL(64,3)-CL(128,3)-CL(256,3)-CL(512,3)-CL(256,3)-CL(128,3)-CL(64,3)-CL(32,3)-CL(16,3)-FCL(1)					
**network_id:**	LSTM1	**layer number:**	12	**parameters number:**	75779817
**training epochs:**	6000	**layer type:**	LSTM Layer(units) ^4^, TimeDistributed Layer(Fully Connected Layer(units))		
**network architecture:**					
LSTM(2048)-LSTM(2048)-LSTM(1024)-LSTM(1024)-LSTM(512)-LSTM(256)-LSTM(128)-LSTM(64)-LSTM(32)-LSTM(16) -LSTM(8)-TDL(FCL(1))					
**network_id:**	LSTM2	**layer number:**	12	**parameters number:**	8523497
**training epochs:**	6000	**layer type:**	LSTM Layer(units), Dropout Layer(dropout_rate), TimeDistributed Layer(Fully Connected Layer(units))		
**network architecture:**					
LSTM(1024)-Dropout(0.2)-LSTM(512)-Dropout(0.2)-LSTM(256)-Dropout(0.2)-LSTM(128)-LSTM(64)-LSTM(32)-LSTM(16) -LSTM(8)-TDL(FCL(1))					
**network_id:**	LSTM3	**layer number:**	12	**parameters number:**	33824489
**training epochs:**	3000	**layer type:**	LSTM Layer(units), Dropout Layer(dropout_rate), TimeDistributed Layer(Fully Connected Layer(units))		
**network architecture:**					
LSTM(2048)-Dropout(0.2)-LSTM(1024)-Dropout(0.2)-LSTM(512)-LSTM(256)-LSTM(128)-LSTM(64)-LSTM(32)-LSTM(16) -LSTM(8)-TDL(FCL(1))					
**network_id:**	LSTM4	layer number:	12	**parameters number:**	44316393
**training epochs:**	6000	**layer type:**	LSTM Layer(units), TimeDistributed Layer(Fully Connected Layer(units))		
**network architecture:**					
LSTM(2048)-LSTM(1024)-LSTM(1024)-LSTM(512)-LSTM(512)-LSTM(256)-LSTM(128)-LSTM(64)-LSTM(32)-LSTM(16) -LSTM(8)-TDL(FCL(1))					

^1^ “Units” represents the activation unit number for this fully connected layer (FCL). ^2^ “Filter” and “kernel_size” represent the number of output filters and the width of the convolution window for this convolutional layer (CL). ^3^ “Pool_size” represents the size of the spatial window for this pooling layer (PL). ^4^ “Units” represents the dimensionality of the output space for this LSTM layer.

**Table A3:** The prediction RMSE for the five testing folds and the results of the Chi-squared test.

The RMSE Value for Each Testing Fold (mg/dL)					
**Models**	**Fold1**	**Fold2**	**Fold3**	**Fold4**	**Fold5**
FCN1	15.96	16.08	16.83	16.46	16.68
FCN2	15.93	16.39	16.88	16.74	16.87
CNN1	15.83	16.12	16.95	16.69	16.07
CNN2	16.03	16.25	16.54	16.02	16.16
CNN3	16.12	16.49	16.88	16.17	16.58
CNN4	16.28	16.00	16.64	16.09	15.90
CNN5	16.09	16.03	16.64	16.59	16.14
CNN6	15.87	16.04	16.71	16.54	16.35
CNN7	15.79	16.22	16.6	16.83	16.16
LSTM1	16.43	16.53	16.66	16.60	16.42
LSTM2	16.67	17.06	17.44	17.06	17.06
LSTM3	16.8	16.81	17.41	17.28	17.46
LSTM4	16.27	16.72	16.86	16.53	16.69
The results for the Chi-Squared Test					
	$\chi^{2}$:		0.1236		
	Freedom degree:		48		
	*p*-value:		>0.995		
